# Drug screening and development cascade for Chagas disease: an update of *in vitro* and *in vivo* experimental models

**DOI:** 10.1590/0074-02760240057

**Published:** 2024-07-01

**Authors:** Maria de Nazaré Correia Soeiro, Policarpo Ademar Sales-Junior, Valeria Rêgo Alves Pereira, Marcos André Vannier-Santos, Silvane Maria Fonseca Murta, Andrea Silvestre de Sousa, Luiz Henrique Conde Sangenis, Alejandro Marcel Hasslocher Moreno, Núbia Boechat, Frederico Silva Castelo Branco, Fabíola Barbieri Holetz, Andrea Rodrigues Ávila, Mirian Claudia de Souza Pereira

**Affiliations:** 1 Fundação Oswaldo Cruz-Fiocruz, Instituto Oswaldo Cruz, Laboratório de Biologia Celular, Rio de Janeiro, RJ, Brasil; 2 Fundação Oswaldo Cruz-Fiocruz, Instituto Aggeu Magalhães, Departamento de Imunologia, Laboratório de Imunopatologia e Biologia Molecular, Recife, PE, Brasil; 3 Fundação Oswaldo Cruz-Fiocruz, Instituto Oswaldo Cruz, Laboratório de Inovações em Terapias, Ensino e Bioprodutos, Rio de Janeiro, RJ, Brasil; 4 Fundação Oswaldo Cruz-Fiocruz, Instituto René Rachou, Belo Horizonte, MG, Brasil; 5 Fundação Oswaldo Cruz-Fiocruz, Instituto Nacional de Infectologia Evandro Chagas, Laboratório de Pesquisa Clínica em Doença de Chagas, Rio de Janeiro, RJ, Brasil; 6 Fundação Oswaldo Cruz-Fiocruz, Instituto de Tecnologia em Fármacos - Farmanguinhos, Departamento de Síntese de Fármacos e Bioativos, Laboratório de Síntese de Fármacos, Rio de Janeiro, RJ, Brasil; 7 Fundação Oswaldo Cruz-Fiocruz, Instituto Carlos Chagas, Laboratório de Regulação da Expressão Gênica, Curitiba, PR, Brasil; 8 Fundação Oswaldo Cruz-Fiocruz, Instituto Carlos Chagas, Laboratório de Pesquisa em Apicomplexa, Curitiba, PR, Brasil; 9 Fundação Oswaldo Cruz-Fiocruz, Instituto Oswaldo Cruz, Laboratório de Ultraestrutura Celular, Rio de Janeiro, RJ, Brasil

**Keywords:** Chagas disease, experimental chemotherapy, Trypanosoma cruzi, in vitro, in vivo

## Abstract

Chagas disease is a tropical neglected disease that affects millions of people worldwide, still demanding a more effective and safer therapy, especially in its chronic phase which lacks a treatment that promotes substantial parasitological cure. The technical note of Romanha and collaborators published in 2010 aimed establish a guideline with the set of minimum criteria and decision gates for the development of new agents against *Trypanosoma cruzi* with the focus on developing new antichagasic drugs. In this sense, the present review aims to update this technical note, bringing the state of the art and new advances on this topic in recent years.

## State of the art on Chagas disease drug development and screening

Chagas disease (CD), a parasitic infection caused by the protozoan *Trypanosoma cruzi*, is recognised as a neglected tropical disease (NTD) by the World Health Organization (WHO), as it affects highly vulnerable populations, and consequently, does not attract substantial attention from pharmaceutical industries in the last century.^1^ This silent disease, endemic in 21 countries in Latin America, has also become an important public health problem in non-endemic countries in North America, Europe, Asia, and Oceania, particularly due to populational migration.^2^ It is estimated that more than 6 million individuals are infected, predominantly chronic cases, less than 10% of which were diagnosed and very few (< 1%) had access to treatment.^3^ The therapy introduced more than five decades ago, based on monotherapy with nitroderivatives, nifurtimox (Nfx) or benznidazole (Bz), has lower efficacy in the later phase of the chronic disease, requires long administration periods and induces adverse reactions that may lead to treatment withdrawal in about 30% of the patients.^4^ Besides, there are naturally resistant parasite strains to both nitroderivative drugs.^5^ ^,^ ^6^ Notwithstanding, the production of these drugs is limited to a few organisations in the world, with a history of global shortages, which puts in risk the population’s access to the medicines.^7^

Before the mid-1990s, there was insufficient evidence to advocate for the use of trypanocidal drugs in the treatment of patients with chronic CD. Etiological treatment was deemed necessary only during the acute phase or in cases of reactivation in chronically immunosuppressed patients. It was not until the early 21st century, a century after the disease’s discovery and three decades after the introduction of the only two effective trypanocidal drugs, nifurtimox and benznidazole, that treatment for chronic patients commenced.^8^ In the past decade, guidelines have been established to standardise the indications for treatment. Currently, trypanocidal treatment is recommended for chronic patients in specific situations, besides all new-borns, children, teenagers, women of childbearing age, and young adults (up to 50 years of age) with the indeterminate form or initial stages of heart disease.^9^ In the chronic phase, administering early etiological treatment, particularly in proximity to the acute infection, yields elevated rates of serological cure. Additionally, this approach may exert a positive influence on the clinical outcomes of individuals infected with *T. cruzi*. It not only diminishes the progression of the disease, but also mitigates the incidence of cardiovascular events.^10^

The development of new drugs more effective and safer against its etiological agent represents a great challenge even 115 years after its discovery by Carlos Chagas. Part of this challenge is related to (i) the complexity of pathophysiology that have not yet been completely elucidated and (ii) the genetic variability of parasite strains [and discrete typing units (DTUs) I-VI and TcBat] and hosts, associated to discrete tissue tropism along the infection and drug susceptibility. Furthermore, another major difficulty is the relative lack of standardisation of effectivity assays for new drug candidates through *in vitro* and *in vivo* studies and their translation into clinical findings. More recently, the use of predictions (*in silico* analysis), artificial intelligence, virtual screening and computer-guided drug design comprise important tools in the endeavour to develop novel drugs.^11^ Also, another key factor is related to the quality of the drug candidate. A low quality of the drug starting point in terms of drug likeness, will lead to higher likelihood of failure during the downstream hit-to-lead and lead optimisation phases, so that the previous correct determination of structural properties that influence the absorption, distribution, metabolism, excretion, and toxicity (ADMET) is increasingly important and current in drug discovery.^12^ Also, unless stability studies on the applied solvent are carried out in advance and guarantee the sustainability of the activity over time, the solubilised test substances (stock solutions) must ideally be prepared immediately before use in the culture medium, and should not be stored, even frozen, and reused, at the risk of chemically degrading and generating false-negatives or false-positives in subsequent stages.^13^ ^,^ ^14^

Due to the still limited knowledge regarding *T. cruzi* genetic and chemical validated targets, the phenotypic cascades still represent the gold standard for CD drug development, allowing activity determination upon developmental forms of the parasite involved in human infection such as intracellular amastigotes and trypomastigotes, even in the absence of previously identified and validated targets.^15^ The phenotypic studies by whole-cell screenings include different methodologies and experimental approaches. For *in vitro* assays, (i) high (HTS) or medium (MTS) throughput screening, (ii) high content screenings (HCS) (determining the parasite load by automated fluorescent microscopic analysis of parasites and host cells usually using DNA fluorescent binders or parasites expressing fluorescent proteins), as well as (iii) the use of different types of mammalian cell cultures infected by the parasites (genetically modified or not) is facilitated as *T. cruzi* infect, differentiate, and replicate in all nucleated host cells.^16^ ^,^ ^17^

Thus, many protocols have been proposed using a wide diversity of cellular models as host cells, as well as *T. cruzi* strains belonging to the different DTUs of the parasite that are clinically relevant for human infections.^18^ Studies have demonstrated that the genetic variability of *T. cruzi* and the source of the host cells can impact the response to drug candidates, as they may exhibit variable outcomes related to susceptibility or resistance.^16^ ^,^ ^19^ These variations in drug response and the lack of technical standardisation of assays can impair reproducibility and translation of *in vitro* to *in vivo* findings and may represent yet another obstacle to be faced to successfully move a preclinical candidate into clinical trials.^20^

The discovery and development of new drugs demands high-cost (U$1-2 billion), long-term (10-15 years) steps, requiring a multidisciplinary team. Natural products, their derivatives and synthetic compounds are sources for the development of new drugs. Besides being active in whole-cell assays, drug candidates should preferably meet different requirements regarding their chemical structure, pharmacological properties, and safety profile among others drug-like characteristics. Among them, those chemical properties related to metabolic stability, aqueous solubility at therapeutic concentrations and lipophilicity to diffuse biological membranes can modulate the bioavailability after oral administration, which is the preferred route of administration, especially for NTD. In this sense, the adequate distribution in the body with efficient delivery to the target tissues in therapeutic concentrations are essential properties to attain specificity and safety properties (high selectivity and potency) and are essential to drug efficacy. Furthermore, in the case of a drug for NTD it is even more desirable for the new candidate to have a simple and optimised synthesis process, which consequently impacts its industrial viability and cost in developing countries. In summary, a novel drug candidate for CD must present equal or higher activity than the reference drugs, be safe (favourable toxicity profile with low or without adverse effects) and display favourable pharmacokinetic and pharmacodynamics profiles.^20^

Given this scenario, the parasitology, medicinal chemistry, clinical and molecular experts of the Oswaldo Cruz Foundation (Fiocruz), which are members from the Translational Program in Chagas Disease (FIOCHAGAS), aimed to update the flowchart and corresponding costs for each step (in dollar) (Figure) previously proposed in the technical note by Romanha and collaborators^21^ taking into consideration the current knowledge about experimental chemotherapy against *T. cruzi* and the recommended characteristics of the “Target Product Profile” (TPP) and “Target Candidate Profile” (TCP) of a new drug for CD.^22^

**Figure:**
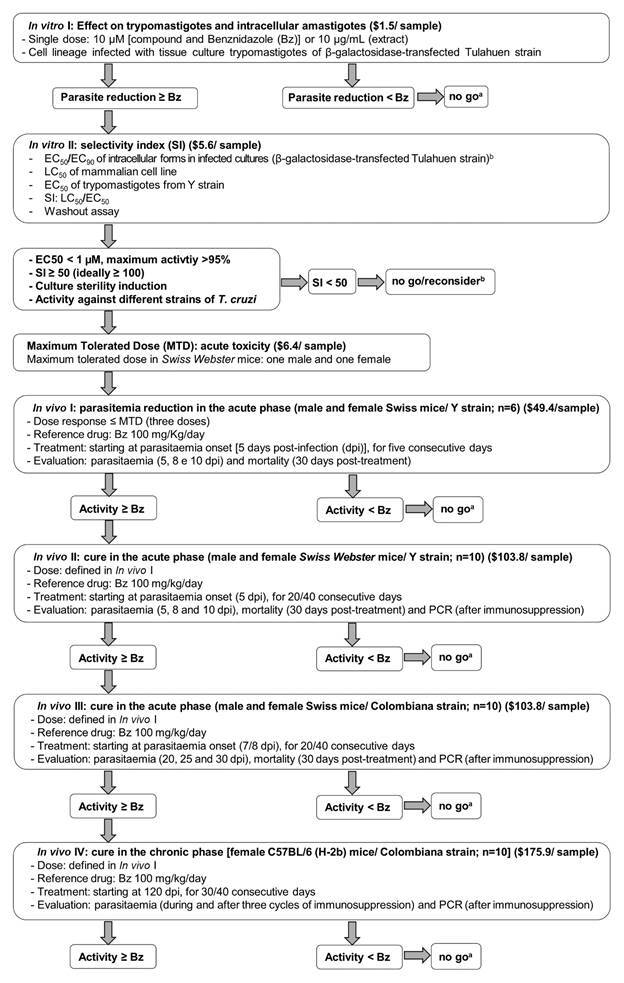
Flowchart for novel drug candidate for Chagas disease. Updated from Romanha et al.^21^

## Drug development for Chagas disease

Based on the potency of Bz (EC_50_ value) on intracellular forms of *T. cruzi* (1 µg/mL = ≈3.8 µM), the flowchart reported by Romanha and collaborators^21^ proposed as a first filter, a cell-based reporter assay using cell lines (such as L929 cell cultures - fibroblasts) infected with Tulahuen strain (DTU VI) transfected to express the β-galactosidase gene with a readout relying on the colorimetric determination of the β-galactosidase activity’ product. In this *in vitro* model, they proposed to start the flowchart assessing a fixed concentration (1 µg/mL for pure synthetic compound and 10 µg/mL for crude extracts or fractions of natural products). Now, based on the current knowledge and updated TCP, the initial step for synthetic and purified compound is proposed using a fixed concentration of 10 µM, which is equivalent to the EC_90_ value of Bz against the intracellular parasites,^23^ while keeping the fixed concentration at 10 µg/mL for crude extracts or fractions from natural sources. At this stage, both parasites genetically modified to express enzymes (β-galactosidase and luciferase) or fluorescent proteins (GFP, RFP, tdTomato, among others) can be used to evaluate the impact of test compounds on the overall parasite load in infected cultures (*e.g.*, L929, Vero, HeLa, L6 lineages, among others).^24^ Only those compounds displaying similar or better activity than Bz undergo a concentration-response curve to define their respective EC_50_ and EC_90_ values.

Regarding toxicity in mammalian cells, unlike the protocol of Romanha and collaborators,^21^ determining the cytotoxicity profile (LC_50_) using a concentration-response curve is recommended, also applying the same cell type employed as host cells, to define the drug selectivity more accurately. Following the current consensus, an active or “hit” compound must ideally reach EC_50_ < 5 µM, maximum activity > 95%, and selectivity indexes (SI) > 10.^25^ Hits can be further evaluated regarding the structure-activity relationship (SAR) studies, undergo structure optimisation, and then be subjected to a new cycle of phenotypic assays aiming to identify a lead compound. A lead compounds for CD ideally should display several characteristics including EC_50_ < 1µM, max activity > 95% and selectivity ≥ 50 among others.^20^

It is also important to highlight additional biological assays to be considered for candidates that exhibit hit profile. These compounds should be assayed for activity against non-dividing and low metabolic forms (trypomastigotes) belonging to different *T. cruzi* DTUs relevant for human infection (*e.g.*, DTU I, II, V and VI), also applying distinct host cell types. Additionally, the drug activity and cytotoxicity assays in primary culture cell models (2D and 3D) are recommended, as they exhibit a better correlation between *in vitro* and *in vivo* findings.^16^ ^,^ ^18^ ^,^ ^26^ ^,^ ^27^ ^,^ ^28^ ^,^ ^29^

Thus, another step to be added to the protocol proposed by Romanha and collaborators^21^ refers to the selection of lead compounds for CD therapy. As endorsed by current literature and above briefly described, lead compounds should exhibit the following characteristics: EC_50_ < 1 µM, maximum activity > 95%, SI > 50 (ideally ≥ 100), be capable of inducing culture sterility (in washout assays), display *in vitro* metabolic stability (rodent and human microsomes), present rapid trypanosomicidal mechanism of action, show activity on different *T. cruzi* strains and DTUs, have solubility at physiological pH (> 50 μg/mL), display free Cmin higher than EC_50_/EC_90_ for ≥ 8 h, low inhibition of human cytochrome P450 (CYP3A4 - IC50 > 10 µM), absence of adverse, mutagenic or genotoxic effects, among other drug likeness profile^24^ Several of these physicochemical and pharmacological properties can be initially predicted by free chemoinformatic servers (*in silico* analysis) and by *in vitro* metabolism models, aiming for a preliminary evaluation of the ADMET profile of the test compound, to move (go-no go) it in proof-of-concept models (*in vivo*). The computational tools, still little explored by many research groups, may contributed to a successful translation between *in vitro* and *in vivo* findings. The data regarding the desirable ADMET and pharmacological parameters are relevant for defining *in vivo* dose-response studies with the preclinical compound candidates.

Recently, quiescent/dormant, and non-replicating parasites have also been implicated in drug resistance, reflecting the low cure efficacy of CD treatment.^30^ Therefore, the development of assays for dormant forms should be a priority in the search for new compounds against CD. Thus, the culture sterility analysis (washout assay) is an additional important approach to be introduced in the technical note proposed by Romanha and collaborators.^21^ This assay allows identifying whether the drug candidates exert a trypanosomicidal or trypanostatic action and their potential effect on “dormant amastigotes” (metabolically quiescent forms).^20^ It represents an important insight to be considered in drug screening studies, considering potential characteristics such as drug resistance and/or subpopulation diversity that may account for parasite recrudescence at the end of the pharmacological pressure.^24^ Washout assays consist of incubating infected cultures with the drug candidates for variable periods, followed by washing and maintenance of the cultures in a compound-free culture medium for a long period (up to 60 days), with daily checking of parasite release into the culture supernatant and monitoring of intracellular parasitism at the endpoint. The parasite recrudescence *in vitro* may be related to the survival and differentiation of persistent forms as well as a static effect that may result in a future therapeutic failure.^26^ ^,^ ^28^ ^,^ ^29^ ^,^ ^31^

We also draw attention to drug combination as it allows, by simultaneously reaching different and/or complementary targets, the reduction of doses, costs, and exposure times, when achieves synergistic or additive effect.^18^ In this regard, among the current clinical trials, one is approaching the synergistic Bz combination with Antabuse (disulfiram), which may pose several advantages^32^ and enhances murine survival.^33^ ^,^ ^34^

Another point to be discussed in the pipeline of drug discovery for CD refers to the recommendation to deprioritise sterol biosynthesis inhibitors, especially the ones that target the 14-α-demethylase (TcCYP51) enzyme. This has been justified by the failure of the repurposed antifungal azoles (posaconazole and fosravuconazole) in chronic CD carriers in clinical trials.^24^ However, this recommendation does not represent a consensus among the different research groups, considering the several limitations observed in the design of the clinical trials^20^ as they were underdosed, and submitted to a very short treatment period, in part due to the high cost of such drugs.^35^ Additionally, as trypanosomatidae possess metabolic differences (and variability) than fungi, additional requirements are needed for novel azole-based drugs to be more efficient, especially related to their pharmacokinetics and tissue distribution profile.^35^ New drugs candidates that are more selective for the trypanosomatid enzyme have been successfully synthetised and some proved to be very active in a *in vivo* model, reaching high parasitological cure rates especially when combined with Bz,^36^ arguing in favour of continuing with studies testing CYP51 inhibitors with greater selectivity to the parasite’s enzyme.^37^ In this sense, we endorse the comment stated by De Rycker^38^ - “*The challenge for drug discovery is to develop compounds that reach all parasite reservoirs and maintain sufficiently high concentrations for long enough to kill all parasites. Importantly, the goal is not to develop drugs for mice but for humans. Understanding of how parasite dynamics and distribution translate to the human patient situation is key, but not easily achieved*”.

Following the flowchart of Romanha and collaborators,^21^ the successful drug candidates in *in vitro* cell-based screening may proceed to *in vivo* proof-of-concept in experimental mouse models of *T. cruzi* infection. Ideally, these drug candidates should be able to significantly suppress the parasitaemia peak at non-toxic doses, up to the maximum tolerated dose (MTD) primarily explored in murine acute toxicity studies. At that time, Romanha and collaborators^21^ proposed the evaluation of the drug candidates mainly in acute models of experimental *T. cruzi* infection using *Swiss Webster* mice infected with the Y strain.^39^ The outbred mouse model infected with this DTU II parasite strain was proposed due to its short-lived acute infection characteristics (*e.g.*, high and rapid parasitaemia onset), enabling a larger window for detection of trypanosomicidal activity evaluated by parasitaemia decline and gain in animal survival, even after cycles of immunosuppression by cyclophosphamide administration after drug treatment.

Now, considering the current knowledge regarding the differential dynamics of host organ/tissue colonisation by *T. cruzi* in the course of infection, chronic models are strongly recommended, since they mimic the indeterminate stage of the disease, when parasitaemia is low, subpatent and intermittent.^40^

In the literature, for both acute and chronic models of experimental CD, various combinations of mouse (inbred/outbred) and *T. cruzi* strains have been used.^24^ In both, the primary outcome is the drug candidate’s ability to achieve sterile cure (elimination of the parasite in all tissues).^18^ ^,^ ^24^ Thus, cycles of mice immunosuppression are still recommended to assess whether treatment prevents parasite recrudescence. The use of genetically modified parasites expressing bioluminescent or fluorescent reporters have allowed real-time monitoring of the dynamics of infection, identifying the persistence of *T. cruzi* in different tissues and organs, thus letting non-invasive assessment of efficacy of drug candidates.^24^ Also, drug intermittent administration has been proposed to achieve parasitological cure even considering the existence of quiescent/dormant parasites.^41^ The authors report that a weekly administration of Bz at a dose 2.5 to 5 -fold the standard daily dose eliminated actively replicating parasites as well as residual, transiently dormant parasite population in mice.^41^

The update of Romanha’s protocol^21^ aims to revise and re-define the minimum flowchart steps to be followed in demonstrating the effectiveness of anti-*T. cruzi* agents by phenotypic screening studies, addressing *in vitro* and *in vivo* procedures, as described below:

(1) *In vitro* models for the discovery and development of new drugs with anti-*T. cruzi* action. This section focuses on the forms and strains of parasites, mammalian cell lines, toxicity tests, automation procedures and definition of cut-off points. Drug efficacy in whole cell-based screening may be explored by multiple tools, including manual quantification of Giemsa-stained cultures in low-throughput assay, colorimetric assay, as a β-galactosidase product, use of bioluminescent and fluorescent *T. cruzi* reporter strains, and furthermore, high content screening (HCS) among other approaches.

(i) First step: as proposed by Romanha and collaborators,^21^ the simultaneous analysis against both amastigotes and trypomastigotes is first evaluated using a single concentration of the compound assessed on a cell line (*e.g.*, Vero, HeLa, L929 and L6) infected by *T. cruzi* under experimental conditions that achieve ≥ 50% parasitism of the host cells.^42^ ^,^ ^43^ To exclude compounds with low potential to eradicate the intracellular parasites, the use of a single fixed concentration against intracellular forms (10 µM for synthetic and purified substances and 10 µg/mL for natural products such as fractions and extracts) allows a large-scale screening in automated tests. Then, the readout is proposed to be performed after 72-96 h of drug exposure that corresponds to the end of the intracellular *T. cruzi* cell cycle for most parasite strains. As example, reported by Romanha and collaborators,^21^ in cultures infected with Tulahuen strain transfected with β-galactosidase, the enzyme activity is measured at 570 nm after addition of chlorophenol red (for details see^21^). In other genetically modified parasites (luminescent and fluorescent reporter genes) the readout will be carried out according to recommended protocol. Compounds with trypanosomicidal action equal to or greater than Bz are selected for the next step.

(ii) Second step: as proposed by Romanha and collaborators,^21^ the compounds selected in the first step are next evaluated by concentration-response assay to determine the EC_50_, using the same experimental model performed in the first step of the screening such as L929 cell lines infected with β-galactosidase-transfected Tulahuen strain^21^ or other luminescent or fluorescent parasites. In parallel, host cells toxicity is performed using cell viability detection protocols (*e.g.*, tests with alamarBlue^®^ or CellTiter Glo^®^) with maximum compound concentrations (serial dilutions) up to their limit of solubility in culture medium. After incubation (under the same time as used in the infected cultures stated in step 1 - at 72 - 96 h), the concentration that reduces cell viability by 50% (LC_50_) is then determined. Thus, the SI can be calculated based on the LC_50_/EC_50_ ratio. As above discussed, those compounds with favourable drug-like profile (at least by *in silico* prediction) and that achieve effect equal to or greater than Bz (ideally EC_50_ < 1 µM, maximum activity > 95%, and SI ≥ 50) are further considered for studies in acute and chronic infection models of CD,^21^ ^,^ ^25^ ^,^ ^44^ as well as assayed in the complementary biological assays described in the third step.

(iii) Third step: drug candidates exhibiting hit/lead profiles should undergo complementary analyses on different strains/DTUs of *T. cruzi*, due to the broad spectrum of resistance/susceptibility to drugs presented by different parasitic populations.^6^ ^,^ ^45^ ^,^ ^46^ ^,^ ^47^ Therefore, it is recommended to evaluate trypanosomicidal activity on intracellular forms from different *T. cruzi* DTUs, especially those relevant for human infection, as well as those that are naturally resistant to Bz and nifurtimox.^48^ Also, it is relevant to screen the compounds upon the infection of different host cell types, as distinct drug outcomes may be achieved depending on the nature and source of the mammalian cells and parasite strain.^6^ ^,^ ^15^ ^,^ ^26^ The use of primary cultures, such as macrophages and 2D and 3D cardiac cultures is largely recommended, also considering differences in susceptibility to toxic drug effects.^16^ ^,^ ^28^ The 3D cell-based drug screening has emerged as a relevant tool for drug discovery since represent more likely the organisation, microenvironment, and physiology of animal tissues, and then exhibit the potential to increase prediction of translational success between *in vitro* and *in vivo* studies.^49^

Regarding activity upon trypomastigotes, the parasite stage that neither divide nor have a long lifespan *in vitro*, this form is still poorly evaluated by research groups but can be screened employing blood or cultured-derived parasites, genetically modified or not, with up to 24 h (37ºC in 5% CO_2_ atmosphere) of drug exposure, since after longer periods there is a drop in parasite viability and/or differentiation to epimastigote-like forms. The analysis on trypomastigotes, a non-proliferative form with low metabolic status as compared to amastigotes, is highly recommended using different strains and DTUs of *T. cruzi* (*e.g.*, Y strain - DTU II, Colombiana, Dm28c and Sylvio X10/1 - DTU I, Tulahuen and CL - DTU VI). As reported for intracellular forms, the analysis of drug effect on trypomastigotes may be performed by different methodologies, such as quantification by light microscopy, through spectrophotometry using untagged parasites (*e.g.*, AlamarBlue^®^, PrestoBlue, CellTiter Glo^®^) as well as using genetically modified parasites (*e.g.*, luciferase tags, parasites expressing fluorescent proteins).^26^ ^,^ ^50^

In all steps, positive and negative controls are always run in parallel, using parasites exposed only to the vehicle used to dilute the test compounds (such as DMSO among others) and parasites treated with reference drugs (Bz and Nifurtimox) for CD. Results are expressed as the difference in percentage reduction between treated and vehicle-treated parasites as recommend by manufacturers and literature protocols. After the analysis, the EC_50_ and EC_90_ values are determined.

(2) *In vivo* models for the discovery and development of new drug candidates for CD: acute and chronic infection models. This section focuses on acute toxicity, mouse and parasites strains, criteria for evaluating parasitaemia reduction, parasitological cure markers and cut-off values. It is outstanding consider that in the case of oral administration, the preferable vehicle is water (if the substance has total aqueous solubility at the concentration applied), or some non-toxic vehicle at doses that necessarily maintains the suspension homogeneous and stable. The suspension/solution must be prepared immediately before administration to animals and cannot be stored unless previous chemical stability studies of the substance in the applied vehicle are conducted.

As described by Romanha and colleagues,^21^ after *in vitro* hit identification (anti-*T. cruzi* activity and selectivity), the selected compounds displaying favourable ADMET properties (at least by *in silico* prediction ) are moved to acute toxicity studies as a first step to exclude toxic agents *in vivo*. Thus, MTD may be performed using one female and one male mouse for each compound, meeting the requirement to reduce, refine and replace the use of animals in toxicity tests (3R), as preconised by the Organisation for Economic Co-operation and Development (OECD) guidelines.^51^ After defining the MTD, sequential tests may progress using non-toxic doses. The criterion for a compound to progress depends on the parallel analysis of the reference drugs, with cut-off of effectiveness equal to and/or greater than Bz. The recommendations for these experimental models are based on the use of mouse lineages and parasite strains that allow the evaluation of the candidate’s activity, using mouse models that mostly mimic the events observed during acute and chronic CD infection, always running in parallel with Bz at the optimal dose (100 mg/kg/day, sid). The steps include the compound ability to reduce the parasitaemia and then to reach parasitological cure in mice with acute experimental infection with *T. cruzi*. Also, now, there is a recommendation to deep explore the potential of the drug candidate to induce parasitological cure in a mouse chronic CD model. As a control, the infected and untreated group should receive the same compound dilution vehicle. The procedures must follow the protocol described below:

First step (*In vivo* I): following adaptations of the protocol described in Romanha and collaborators,^21^ the first stage allows the analysis of the effect of the drug candidate on the parasite load, using female and male *Swiss Webster* mice (18-20 g, 5-6 animals/ group) infected with 10^4^ Y strain bloodstream trypomastigotes. The animals are treated with three doses, with the highest dose set at the MTD value. Treatment is administered (orally (preferable) or intraperitoneally - ip) for five consecutive days; starting at the parasitaemia onset (in this experimental model between four-five days post-infection (dpi)). Only animals with positive parasitaemia are used, evaluating the following parameters: (a) parasitaemic levels measured microscopically^46^ at 5, 8 and 10 dpi and (b) mortality daily checked up to 30 days after the end of treatment. Both parameters are compared to those achieved with the same treatment protocol with 100 mg/kg/day Bz (given orally, sid). In Romanha’s protocol,^21^ the Y strain was chosen due to its moderate resistance to Bz and Nifurtimox,^6^ and as it has been widely used for *in vitro* and *in vivo* drug activity studies, which may be useful to compare the efficacy of several compounds. Also, this model allows identifying the optimal dose for the subsequent steps of the screening process in a short time frame (the *in vivo* assay can be completed in less than 40 days).

The second step (*In vivo* II) aims to analyse the parasitological cure during the acute phase of the infection (*e.g.*, Y strain). It is recommended female and male *Swiss Webster* mice, 18-20 g, 10 animals/group, infected with 10^4^ bloodstream trypomastigotes and treated with the dose previously established in the *in vivo* stage I. Only animals with positive parasitaemia are used. The drug treatment is administered (orally - preferable, or intraperitoneally) for 20-40 consecutive days, starting at positive parasitaemia (in this specific experimental model at 4-5 dpi). The parameters evaluated to determine the percentage of parasitological cure are: (a) parasitaemia at 5, 8 and 10 dpi, (b) mortality at 30 days after the end of treatment and (c) polymerase chain reaction (PCR)^52^ after immunosuppression at 30 days post-treatment, as previously established.^53^

If the performance of a given compound is like/equal to or better than Bz (100 mg/kg/day, given orally, sid) using the same treatment protocol, it may advance to the next stage of *in vivo* testing. PCR was selected due to its sensitivity^54^ ^,^ ^55^ ^,^ ^56^ and the use of cyclophosphamide results in increased sensitivity to parasitic recrudescence and high levels of parasitaemia.^53^ ^,^ ^54^ ^,^ ^57^

The protocol for the third step (*In vivo* III), aims to evaluate parasitological cure during the acute phase of infection caused by another parasite strain (such as the Colombiana strain which is naturally resistant to Bz) and uses female and male *Swiss Webster* mice (18-20 g, 10 animals/group), intraperitoneal (i.p.) infected with 10^4^ bloodstream trypomastigotes and treated with the dose established in the previous *in vivo* approaches. Only animals with positive parasitaemia are used. Treatment is administered (orally or intraperitoneally) for 20-40 consecutive days, starting at 7-8 dpi, which corresponds, in this experimental model, to the onset of parasitaemia. Parasitaemia will also be assessed at 20, 25 and 30 dpi and mortality recorded 30 days after the end of treatment. Animals that do not show reactivation of parasitaemia will be subjected to immunosuppression with three cycles of cyclophosphamide administered in doses of 50 mg/kg of body weight for four consecutive days with three-days intervals between cycles. Parasitaemia will also be assessed during this procedure, as well as in the two weeks following the end of treatment, which will be determined based on negative PCR results for parasitaemia.^55^ Also, some authors also employ the haemoculture protocol to inspect parasitaemia relapse in experimental models. Briefly, thirty days after the end of long-term treatment, mice are bled from the submandibular vein under anaesthesia and subsequent euthanasia and 0.6 ml of the collected blood is divided into two tubes containing 5 mL of liver infusion triptose (LIT) medium. The tubes are incubated at 28ºC for 30-60 days and examined microscopically for the presence of parasites. If both haemocultures are negative, the mouse is considered cured.^58^ The compound will be approved if it presents results equal to or better than Bz treatment (100 mg/kg/day, orally, sid).

After these stages of studies in acute models, drug candidates with action equal to or greater than Bz are analysed in chronic models. Thus, following a recently established protocol,^59^ female C57BL/6 (H-2b) mice (five-seven weeks old) are infected (i.p. route) with 100 bloodstream trypomastigotes of the Colombiana strain of *T. cruzi*. Since only animals with positive parasitaemia should be used, infection should be confirmed at 7-8 dpi. After 120 days post-infection (dpi), animals in the chronic phase are treated (orally - preferable, or i.p.) with doses established in the previous stages. The animals are subjected to immunosuppression by cyclophosphamide (*e.g.*, three cycles with 50 mg/kg of body weight for four consecutive days and three-days intervals between each cycle). During this procedure, parasitaemia relapse will be assessed by light microscopy (and/or by imaging analysis), as well by PCR at the end of treatment.^55^

## Concluding remarks

Chagas disease caused by the protozoan *T. cruzi* still represents a serious silent public health illness mostly afflicting neglected populations in the poorest areas of the tropical and subtropical countries, lacking vaccine, safer and more effective alternative therapies. Due to the low interest of most pharmaceutic industries associated to the disease complexity, fewer drugs were moved to clinical trials and up to now none with better efficacy than the one of reference drug (benznidazole). Thus, presently an update to the technical note reported in 2010 by Romanha and collaborators^21^ was performed aiming to collaborate to the standardisation of *in vitro* and *in vivo* protocols to identify new drug candidates that could be used in the future clinical studies with improved chances of success.
