# ERRATUM

**DOI:** 10.1590/0074-02760240057er

**Published:** 2024-07-29

In the article “D**rug screening and development cascade for Chagas disease: an update of *in vitro* and *in vivo* experimental models**”, DOI number: 10.1590/0074-02760240057, published in *Mem Inst Oswaldo Cruz*, Rio de Janeiro, Vol. 119: e240057, 2024, on page 1 (Authors’ name):

Where it reads:

Alejandro Marcel Hasslocher Moreno

It should read:

Alejandro Marcel Hasslocher-Moreno

